# Perturbations of the actin cytoskeleton activate a *Dictyostelium* STAT signalling pathway

**DOI:** 10.1016/j.ejcb.2012.01.002

**Published:** 2012-05

**Authors:** Tsuyoshi Araki, Jeffrey G. Williams

**Affiliations:** College of Life Sciences, Welcome Trust Biocentre, University of Dundee, Dow St., Dundee DD1 5EH, United Kingdom

**Keywords:** *Dictyostelium*, STAT, Hyper-osmotic stress, DIF-1, Actin cytoskeleton

## Abstract

The *Dictyostelium* transcription factor STATc is tyrosine phosphorylated and accumulates in the nucleus when cells are exposed either to hyper-osmotic stress or to the prestalk-inducing polyketide DIF-1. In the case of stress STAT activation is mediated by regulated dephosphorylation; whereby two serine residues on PTP3, the tyrosine phosphatase that de-activates STATc, become phosphorylated after exposure to stress so inhibiting enzymatic activity. We now show that the more highly regulated of the two PTP3 serine residues, S747, is also phosphorylated in response to DIF-1, suggesting a common activation mechanism. Hyper-osmotic stress causes a re-distribution of F-actin to the cortex, cell rounding and shrinkage and we show that DIF-1 induces a similar but transient F-actin re-distribution and rounding response. We also find that two mechanistically distinct inhibitors of actin polymerization, latrunculin A and cytochalasin A induce phosphorylation at S747 of PTP3 and activate STATc. We suggest that PTP3 phosphorylation, and consequent STATc activation, are regulated by changes in F-actin polymerization status during stress and DIF-induced cytoskeletal remodelling.

## Introduction

Metazoans STATs are activated as transcription factors and accumulate in the nucleus when a cytokine binds to its receptor (reviewed by Darnell, 1997). Activation is effected by tyrosine phosphorylation at a single site and the activating tyrosine kinase is, most frequently, a member of the JAK family. An alternate, and much less well understood, pathway of activation for STAT1 and STAT3 is triggered by hyper-osmotic stress (Gatsios et al., 1998). This induces tyrosine auto-phosphorylation of Jak1, Jak2 and Tyk2 and activates the two STATs. Tyk2 and Jak2 are not required for stress-induced STAT activation but Jak1 is essential for a full response.

While the physiological role of hyper-osmotic stress induced activation is unclear for the metazoan STATs, hyper-osmotic activation of *Dictyostelium* STATc is known to be essential for the induction of approximately 20% of stress activated genes (Araki et al., 2003; Na et al., 2007). The mechanism of STATc activation is partially understood. There is regulation at the level of tyrosine phosphorylation; exerted by an as yet unidentified tyrosine kinase, utilizing cGMP as a second messenger and requiring the ROCO family member GbpC (Araki et al., 2010). The unidentified activating tyrosine kinase functions synergistically with a regulated de-phosphorylation reaction, effected by the soluble protein tyrosine phosphatase PTP3 (Araki et al., 2008). There are two sites of serine phosphorylation on PTP3, S448 and S747, and modification at these sites correlates with a reduction in phosphatase activity. Hyper-osmotic stress induces phosphorylation at both sites but there is a much higher induction ratio at S747. We show here that the activating ligand for STATc, DIF-1, also directs phosphorylation at S747.

DIF-1 is a chlorinated hexaphenone, produced by the prespore cells, that directs prestalk differentiation (Kay and Thompson, 2001). Although several transcription factors respond to DIF-1 by accumulating in the nucleus the upstream steps in their activation are unknown (reviewed by Williams, 2006). The upstream steps in the stress-induced modification of PTP3 are also largely unknown, although pharmacological agents that increase cytosolic calcium levels stimulate phosphorylation of PTP3 and so activate STATc (Araki et al., 2010). Here we show that manipulations that affect the actin cytoskeleton also lead to STATc activation and that activation correlates with increased phosphorylation of PTP3 on S747. We suggest a causal relationship between PTP3 phosphorylation and remodelling of the actin cytoskeleton.

## Results

### DIF-1 induces phosphorylation of PTP3 and causes a transient remodelling of cell shape and the actin cytoskeleton

**Fig. 1:**
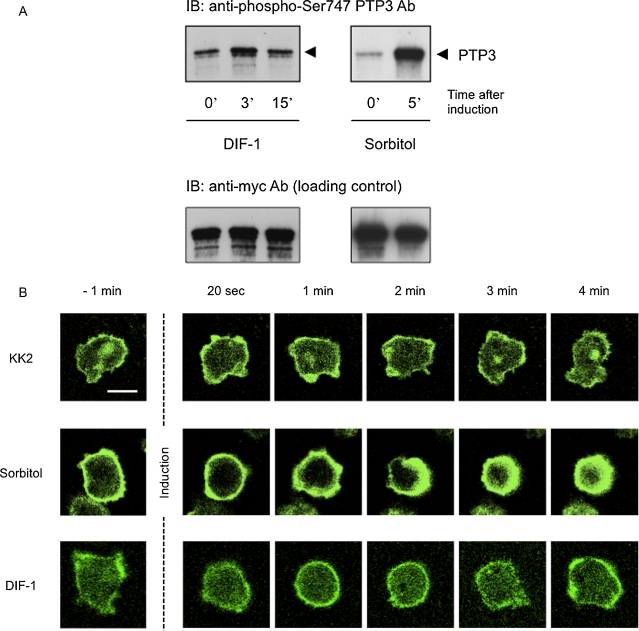

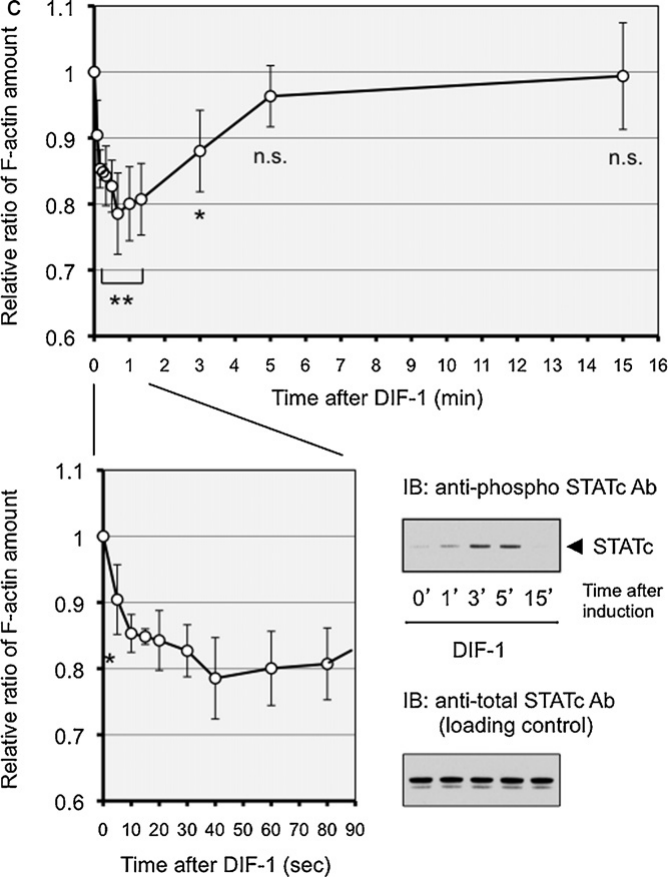
(A) Induction of PTP3 phosphorylation by DIF-1. Cells transformed with mycPTP3ΔCS, a fusion of a myc epitope tag to the N terminus of PTP3 (Araki et al., 2008), were developed in suspension in KK2 buffer (20 mM K_2_HPO_4_/KH_2_PO_4_ pH 6.2) for 4 h. The cells were then incubated in KK2, or the same buffer containing sorbitol at 200 mM or DIF-1 at 100 nM, for the indicated times. The degree of phosphorylation at Ser 747 of PTP3 was measured by Western transfer, using a phospho-specific antibody. The blot was re-probed with the 9E10 myc antibody to provide loading controls. This is a typical result from three independent experiments. (B) Induction of cell rounding and actin re-organisation by DIF-1. Cells transformed with ABP:GFP, a fusion of the ABP120 F-actin binding protein fragment to a C-terminally located GFP reporter, were developed in suspension in KK2 buffer for 3.5 h. The cells were plated at a density of 1.5 × 10^5^ cells/cm^2^ on membrane-bottomed dishes (petriPERM50 hydrophilic, VIVASCIENCE). After 30 min, the cells were incubated in KK2 or the same buffer containing sorbitol at 200 mM or DIF-1 at 100 nM. Individual cells were imaged in an inverted confocal microscope (Leica DMRBE model SP2) at the indicated times thereafter. These are single images from a Z series. This is a typical result from three independent experiments. The scale bar represents 10 μm. (C) Induction of transient actin de-polymerisation by DIF-1. Cells were developed in suspension in KK2 for 4 h. They were immediately lysed in Triton X-100 or incubated in DIF-1 at 100 nM for the indicated times and then lysed. After centrifugation the pellets were analysed by SDS gel electrophoresis and the amount of F-actin, determined by scanning, was plotted relative to that in the zero time control. This is the mean ± SD of four independent experiments. The inset at the lower left is a blow up of the early part of the induction. Student's *t*-test showed a *P* value of <0.01 for a difference from the zero time value at all the early time points (indicated by the symbol ** in the main figure), except for the 5 s point. Here the value was <0.05 (indicated by the symbol * in the inset). The panel at the lower right shows the activation kinetics for STATc.

We have previously shown that sorbitol treatment induces a small increase in phosphorylation of PTP3 on S448 and a much larger increase in phosphorylation on S747 (Araki et al., 2010). The results in Fig. 1A show that DIF-1 also directs increased phosphorylation at S747. In this particular experiment there was a small increase in phosphorylation of S448 after DIF-1 treatment (data not shown) but this was not consistently observed and S448 phosphorylation was not further investigated.

The data in Fig. 1B prove that the two inducers share another property; both induce rounding of the cell and restructuring of the actin cytoskeleton to form a cortical shell. Just as with the activation of STATc (Araki et al., 2003), the DIF-1 induced actin re-organisation is significantly weaker and more transient than that of sorbitol. The cell remains in this state for only one or 2 min. *Dictyostelium* cells also show a transient rounding and actin re-modelling when exposed to cAMP and, during this “cringe” response (Futrelle et al., 1982), there is a transient increase in the amount of F-actin associated with the Triton X-100 insoluble cytoskeleton (McRobbie and Newell, 1983). Surprisingly, the converse holds true for the DIF-induced cringe response; here there is a transient reduction in the amount of F-actin associated with the insoluble cytoskeleton (Fig. 1C).

### Pharmacological disruption of the actin cytoskeleton activates STATc

**Fig. 2:**
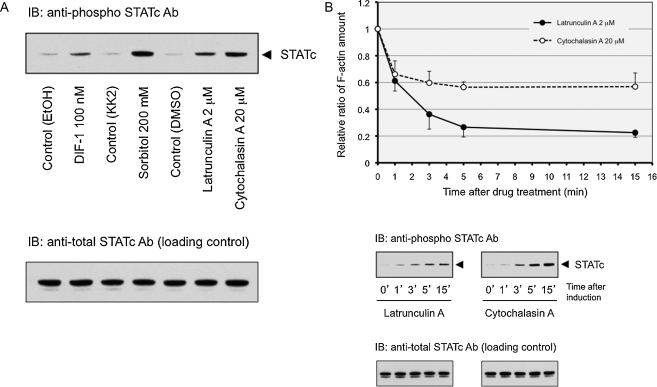
(A) Activation of STATc by drugs that perturb the actin cytoskeleton. Cells were developed in suspension for 4 h and then left untreated or treated with the indicated agents (DMSO is included as it is the vehicle for latrunculin A and cytochalasin A). After 5 min samples were lysed and STATc tyrosine phosphorylation was determined as described in “Methods” section. This is a typical result from one of four independent experiments. (B) Comparison of the kinetics of actin de-polymerisation and activation of STATc by drugs that perturb the actin cytoskeleton. Cells were developed in suspension for 4 h and then left untreated or treated with latrunculin A and cytochalasin A for the indicated times. One set of samples was analysed for cytoskeletal F-actin as in Fig. 1C. This graph shows the mean ± SD of four independent experiments. Another set of samples was lysed and STATc tyrosine phosphorylation was determined as in (A). This is a typical result from one of the four independent experiments.

The above results suggest a potential involvement of the actin cytoskeleton in STATc activation and this possibility was tested using two drugs that disrupt the cytoskeleton: latrunculin A and cytochalasin A. Both induce STATc tyrosine phosphorylation, with cytochalasin A acting as the more efficient inducer (Fig. 2A). We also compared the kinetics of F-actin dissociation and STATc tyrosine phosphorylation using the two drugs. Both induce F-actin dissociation with similar kinetics; a decline in F-actin is detectable after just 1 min of treatment and it plateaus by about 5 min (Fig. 2B). These are very similar kinetics to that of STATc activation, determined in parallel (Fig. 2B).

### Pharmacological disruption of the actin cytoskeleton induces nuclear translocation of STATc

**Fig. 3:**
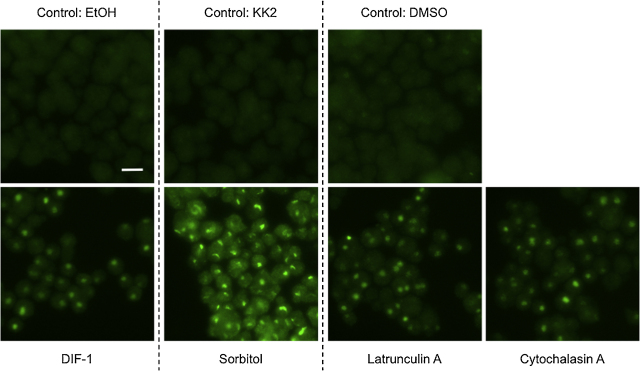
Nuclear translocation of STATc in response to latrunculin A and cytochalasin A. Cells were developed in suspension for 4 h and then treated with the indicated agents or their vehicles. After 5 min the samples were fixed and STATc localisation was determined as described in “Methods” section. This is a typical result from one of the four experiments described in Fig. 2A. The scale bar represents 10 μm.

Using both latrunculin A and cytochalasin A, we confirmed that increased tyrosine phosphorylation of STATc leads to increased STATc nuclear accumulation (Fig. 3). There is, however, an apparent anomaly with sorbitol; which was included as a positive control. Sorbitol and cytochalasin induce approximately equal amounts of tyrosine phosphorylation of STATc but sorbitol is a stronger inducer of STATc nuclear accumulation than is cytochalasin A. Perhaps, therefore, osmotic stress signals through an additional, parallel pathway, that is independent of tyrosine phosphorylation, to stimulate the nuclear translocation of STATc.

### Disruption of the actin cytoskeleton causes increased serine phosphorylation of PTP3

**Fig. 4:**
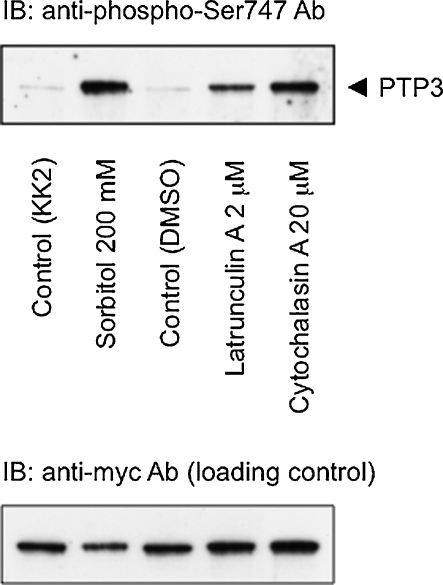
Specific phosphorylation of PTP3 in response to latrunculin A and cytochalasin A. Cells were developed in suspension for 4 h and then left untreated, or treated with the indicated agents (DMSO is included because it is the vehicle for latrunculin A and cytochalasin A). After five min. samples were lysed and the degree of serine phosphorylation of PTP3 at Ser 747 was analysed as described in “Methods” section. This is a typical result from one of four independent experiments.

The phosphorylation status of PTP3 was monitored in latrunculin A and cytochalasin A-treated cells, using the S747 phospho-specific antibody. Both drugs induce an increase in phosphorylation at S747 but, as with STATc activation, there is a larger response with cytochalasin A (Fig. 4).

## Discussion

The demonstration that DIF-1 treatment leads to increased phosphorylation at S747 of PTP3 reveals an additional convergence point for the developmental, DIF-1 signalling pathway and the cell protective, hyper-osmotic stress response pathway. Another point of similarity is that sorbitol and DIF-1 both induce cellular rounding and cortical accumulation of F-actin. Such a response has not to our knowledge been previously directly documented for DIF-1; although oscillatory light scattering changes, induced by endogenously generated cAMP pulses, are phase-shifted in the presence of DIF-1 and such a shift is symptomatic of a cellular shape change (Wurster and Kay, 1990). The magnitude and duration of the cell shape and actin cytoskeletal responses are very different for DIF-1 and sorbitol and this correlates well with the different activation kinetics of the two STATc inducers. Both show a similarly rapid rise in STATc activation but the DIF-1 response peaks at 1–2 min and then falls rapidly while with sorbitol STATc is activated to a higher level and activation is maintained for as long as sorbitol is present (Araki et al., 2003).

Exposure to DIF-1 also causes a transitory decline in the amount of F-actin associated with the cytoskeleton. We can analyse cytoskeletal associated F-actin very rapidly, using direct cell lysis, and find a statistically significant drop in its amount after just 5 s. The minimal level is reached by about 1 min and recovery is effected by about 5 min. STATc activation is detectable after 1 min of DIF treatment (Fig. 1C and Araki et al., 2003; this is the earliest time point analysable because, to obtain reproducible results, the cells must be harvested by centrifugation before lysis). The peak is at 3 min and tyrosine phosphorylation of STATc declines thereafter. Thus there is an approximate gap of 2 min between the maximal dissociation of F-actin and maximal STATc activation. This perhaps reflects a requirement for an intermediate step, such as the recruitment of a signalling complex.

Pharmacological disruption of the actin cytoskeleton, by exposure to either one of two drugs, also causes robust activation of STATc. The telling fact is that, over the 15 min period analysed, both drugs induce a plateau level of F-actin dissociation and a plateau level of STATc activation. This contrasts markedly with the highly transitory effects of exposure to DIF-1. Thus there is a temporal correlation, for all three agents, between their individual kinetics of F-actin dissociation and their ability to activate STATc. The one unpredicted feature of the drug kinetic data is that the plateau level of F-actin dissociation for cytochalasin A is significantly higher than for latrunculin A while, conversely, the plateau level of STATc activation is reproducibly higher for cytochalasin A. There are several potential explanations for this apparent non-congruence. There may be a side-effect of one or other of the two drugs or there may be a physiologically relevant difference between the two; as we do not know the precise mechanism of the STATc activation engendered by dissociation of the actin cytoskeleton and the two drugs act to bring about dissociation by very different mechanisms. Latrunculin A binds actin monomers and prevents them from polymerizing while cytochalasin A binds to microfilaments and prevents them extending.

We also investigated the mechanism of STATc activation and showed that both pharmacological agents cause increased phosphorylation at residue S747 of PTP3; suggesting that activation occurs by inhibition of phosphatase activity. How might such an inhibition be effected? The observations could be reconciled if the inhibition of PTP3 activity was caused by G-actin, or by F-actin oligomers, generated during re-modelling of the cytoskeleton or by exposure to pharmacological agents. The latter would include latrunculin A and cytochalasin A but might also include agents, such as BHQ and thapsigargin that liberate calcium into the cytosol. Such agents stimulate phosphorylation at S448 and S747 of PTP3 and activate STATc (Araki et al., 2010) but they could exert this effect indirectly, via calcium-regulated cytoskeletal modulators (Schleicher et al., 1990; Furukawa et al., 2003). There is one very well understood precedent for G-actin functioning as an indirect regulator of transcription (reviewed by Olson and Nordheim, 2010). G-actin binds to and sequesters myocardin-related transcription factor (MRTF) cofactors in the cytoplasm. When G-actin levels fall, as a result of actin polymerization, MRTFs are liberated to accumulate in the nucleus where they bind to SRF (Serum Response Factor). The MRTF–SRF complex then activates expression of the sub-set of SRF target genes that encode cytoskeletal proteins.

Hyper-osmotic stress causes phosphorylation of *Dictyostelium* actin on tyrosine-53 and over-expression of a non-phosphorylatable, Y to A, mutant form of actin causes fragmentation of the actin cytoskeleton (Shu et al., 2010). Hence one possibility we considered is that the tyrosine phosphorylation of actin might be an upstream step in the PTP3-STATc activation pathway. We attempted to test this idea, by searching for constitutive STATc tyrosine phosphorylation in an over-expressor strain for the Y53A mutant form of actin. Although we failed to detect this (unpublished results), a negative result in such an experiment is very difficult to interpret and it remains a possibility.

We have previously provided evidence that the ROCO family kinase GbpC lies in the signalling cascade that activates the kinase, responsible for tyrosine phosphorylating STATc (Araki et al., 2010). It has now been shown that osmotic stress causes GbpC to move from the cytosol to associate with the cell cortex and that latrunculin A treatment prevents this re-distribution (Kortholt et al., 2012). Although this implicates the tyrosine kinase arm of the proposed dual control system rather than the tyrosine phosphatase arm (Araki et al., 2010), these observations provide additional support for an involvement of the actin cytoskeleton in STATc activation.

While these various pieces of evidence imply some kind of causal relationship between changes in the actin cytoskeleton and STATc activation this is almost certainly not the entire explanation. When cAMP is added to cells it causes a cringe response, very similar to that induced by DIF-1 but with a transient increase in F-actin associated with the detergent-insoluble actin cytoskeleton rather than the decrease observed with DIF-1. Also, cAMP does not induce activation of STATc (Araki et al., 2003) but it does cause translocation of GbpC to the cortex (Kortholt et al., 2012). Both DIF-1 and hyper-osmotic stress presumably, therefore, induce other changes that are also essential for STATc activation.

## Materials and methods

### Cell culture and development

*Dictyostelium* strain Ax-2 (Gerisch isolate) cells were grown axenically in HL-5 medium (Watts and Ashworth, 1970). For induction cells were resuspended in KK2 buffer (16.1 mM KH_2_PO_4_, 3.7 mM K_2_HPO_4_ pH 6.2) at a concentration of 1 × 10^7^ cells/ml and shaken for 4 h at 200 rpm. Sorbitol was added to a final 200 mM, DIF-1 to 100 nM, latrunculin A to 2 μM and cytochalasin A to 20 μM.

### Immuno-chemical analyses

Polyclonal, phospho-specific PTP3 and monoclonal STATc (CP22) antibodies were used in Western transfer as described previously (Araki et al., 2010). In the case of STATc immuno-staining was performed using the non phospho-specific monoclonal antibody 7H3.

#### Association of F-actin with the detergent-insoluble cytoskeleton

Determination of F-actin levels was performed using a slightly modified method from that originally described (McRobbie and Newell, 1983). DIF-1 treated cells in suspension (1 × 10^7^ cells/ml) were lysed by adding 2× Triton lysis buffer (2% Triton X-100, 20 mM immidazole, 20 mM EGTA and 4 mM sodium azide). The cell lysate was incubated on ice for 10 min and then at 21 °C for 10 min. The lysate was spun at 11,000 × *g* for 4 min and the pellet was washed once in 1× Triton lysis buffer. This pellet was deemed the Triton X-100 insoluble fraction and was analysed by SDS-PAGE followed by Colloidal Blue staining. Quantification of actin was performed by gel scanning and analysis using ImageJ software.
