# Supplementary material for: Perturbations of the actin cytoskeleton activate a Dictyostelium STAT signalling pathway
Source: Eur J Cell Biol. 2012 May;91(5):420–5. doi: 10.1016/j.ejcb.2012.01.002 (PMC3315007; doi:10.1016/j.ejcb.2012.01.002)
Supplement: Supplementary file 1 [file mmc1.doc]

Ms. Ref. No.: EJCB-D-11-00162
Title: Perturbations of the actin cytoskeleton activate a Dictyostelium STAT signalling pathway
European Journal of Cell Biology

| Received | Oct 05, 2011 |
| --- | --- |
| Decision | Nov 16, 2011 |
| Revision received | Dec 20, 2011 |
| Accepted | Jan 05, 2012 |

**Decision letter**

Dear Dr. Williams,

Your manuscript has now been seen by two referees whose comments are enclosed. Both reviewers raise a number of points that preclude me from accepting your manuscript at this point. A major question that must be clarified is the causal relationship between DIF1-induced destabilization of F-Actin and STATc activation, and reviewer 1 suggests a way to do this. If you think you can satisfactorily answer this criticism as well as all other queries of the reviewers, I should be glad to consider your manuscript further.

Please submit a list of changes or a rebuttal against each point which is being raised when you submit the revised manuscript.

In addition, please prepare the reference list more closely to EJCB style, i.e. all initials after the names. If you use a reference managing software, the output style for J. Microbiol. Methods fits exaclty, but that for J. Cell Sci. would also suffice.

Yours sincerely,

Manfred Schliwa
Editor


p.p. Dagmar Gebauer
Editorial Office
European Journal of Cell Biology

Reviewers' comments:

Reviewer 1

Authors discovered that DIF-1 treatment not only activates STATc, but also induces transient decrease in the cellular level of F-Actin, which is distinct from the previously known hyperosmotic stress induced STATc activation with concomitant increase in F-Actin level. It seems possible, as the authors argued, that DIF-1 induced transient destabilization of F-Actin may function as an upstream event of STATc activation, but it is also possible that the two events are simply coincidental. Unless authors show that DIF-1 mediated STATc activation requires transient decrease in F-Actin level, it remains a weak argument and thus a major problem. One of the possible ways to support the argument would be blocking the transient F-Actin decrease. If STATc activation is compromised in the presence of F-Actin stabilizer, then the argument will be a stronger one. The authors need to respond to the above-mentioned main problem as well as the following minor points.    

There are also some minor points:

1. F-Actin levels decreased in one to two minute after DIF-1 treatment, but STATc activation persisted even when F-Actin level recovered.  Need to explain this.

2. Does persistent F-Actin destabilization by inhibitors activate STATc with similar kinetics? A figure similar to Fig 1C showing F-Actin level vs. time after inhibitor treatment will allow one to better see how closely those two events are related.  

3. cAMP induced F-Actin activation peaks around 10-20 seconds which adapts by 1 minute. How does authors exclude that there is no increase in F-Actin level by DIF-1 treatment?

4. Fig 1B. There is no image for the time zero. Need to show how cells were before the treatments.

5. Fig 1B. Cell cringing effect (at 1 and 2 minute) from DIF-1 is less than impressive and there seems to be a second peak at 4 minute. Need either quantification or better images.


Reviewer 2
Araki and Williams straightforward short communication demonstrates that phosphorylation of PTP3 could be induced by DIF-1, which also caused transient cytoskeleton remodelling, STATc phosphorylation and nuclear translocation. Interestingly, cytoskeleton perturbations by themselves led to STATc and PTP3 phosphorylation.

Specific points:

Figures (or M&M) need to mention how many times experiments have been done;


Fig. 1C which mentions that it represents 3 independent experiments needs p values - this could then be included in the Results section (p.3, §2) where it is mentioned the decrease in the amount of actin associated with insoluble cytoskeleton;


Despite a convincing p-signal, Fig. 2A needs the loading control (e.g. STAT);


Fig. 2A and 2B: assuming that all samples in 2A reflect an equal loading, it would appear that despite equivalent amounts of p-STAT in sorbitol and cytochalasin A-treated samples, there is much more nuclear p-STAT in the nucleus after sorbitol treatment than cytochalasin A. This suggests that perturbations in the cytoskeleton are not sufficient for nuclear translocation of STAT, or alternatively, nuclear retention. The authors should include this in the discussion;


There are a few typos in M&Ms and legends that should be corrected
---------------------------------------------------------------------------------------------------------------------------------------------------------

**Authors response letter**

The detailed responses to the reviewers' comments are below but important

support for an involvement of the actin cytoskeleton in STATc activation derives

from a paper just published by van Haastert’s group (Kortholt et al., 2011). We

therefore now include a description of these results.

P 6 line 30 “We have previously produced evidence that the ROCO family kinase

GbpC lies in the signalling cascade that activates the kinase, responsible for tyrosine

phosphorylating STATc (Araki et al., 2010). It has now been shown that osmotic

stress causes GbpC to move from the cytosol to associate with the cell cortex and that

latrunculin A treatment prevents this re-distribution (Kortholt et al., 2011). Although

this implicates the tyrosine kinase arm of the proposed dual control system rather than

the tyrosine phosphatase arm (Araki et al., 2010), these observations provide support

for an involvement of the actin cytoskeleton in STATc activation”.

Reviewer 1

*One of the possible ways to support the argument would be blocking the transient FActin*

*decrease. If STATc activation is compromised in the presence of F-Actin*

*stabilizer, then the argument will be a stronger one.*

We agree with the reviewer that this would be a valuable way to complement our

demonstration that disruption of the actin cytoskeleton activates STATc and we have

made repeated attempts to do this over several years. These used jasplakinolide,

because this is the most widely used stabiliser and there is a previous report from

Knecht’s group that jasplakinolide stabilises Dictyostelium F-Actin (Lee et al., 1998).

Unfortunately none of our experiments, which used three different batches of the drug

(Knecht reports varying potency of different batches), showed an increase in F-Actin.

This could be a strain difference, Knecht used Ax4 we used Ax2. Or it could reflect a

difference in cellular status, Knecht grew cells on coverslips while we used cells

starved in suspension for four hours. Whatever the explanation we are unable,

unfortunately, to use this approach to bolster the evidence for an interaction with

actin.

*There are also some minor points:*

*1. F-Actin levels decreased in one to two minute after DIF-1 treatment, but STATc*

*activation persisted even when F-Actin level recovered. Need to explain this.*

This should have been discussed and is now done so in the context of the new more

detailed time course presented in Fig 1C.

P5 line 6 “Exposure to DIF-1 also causes a transitory decline in the amount of F-actin

associated with the cytoskeleton. We can analyse cytoskeletal associated F-actin very

rapidly, using direct cell lysates, and find a statistically significant drop in its amount

after just five seconds. The minimal level is reached by about one minute and

recovery is effected by about five minutes. STATc activation is detectable after one

minute of DIF treatment (Fig 1C and Araki et al., 2003; this is the earliest time point

analysable because, to obtain reproducible results, the cells must be harvested by

centrifugation before lysis). The peak is at three minutes and tyrosine phosphorylation

of STATc declines rapidly thereafter. Thus there is an approximate two minute gap

between the maximal dissociation of F-Actin and maximal STATc activation. This

perhaps reflects a requirement for an intermediate step, such as the recruitment of a

signalling complex”.

*2. Does persistent F-Actin destabilization by inhibitors activate STATc with similar*

*kinetics? A figure similar to Fig 1C showing F-Actin level vs. time after inhibitor*

*treatment will allow one to better see how closely those two events are related.*

We have now done just as the reviewer requested and the results nicely support a

temporal link for both drugs. We greatly appreciate the suggestion. The data is

presented as new Fig 2B and in:

Results section: p 3 line 33:

“We also compared the kinetics of F-actin dissociation and STATc tyrosine

phosphorylation using the two drugs. Both induce F-actin dissociation with similar

kinetics; a decline in F-actin is detectable after just one minute of treatment and it

plateaus by about five minutes (Fig 2B). These are very similar kinetics to those of

STATc activation, determined in parallel (Fig 2B).”

Discussion section p5 line 18:

“Pharmacological disruption of the actin cytoskeleton, by exposure to either one of

two drugs, also causes robust activation of STATc. The telling fact is that, over the

15minute period analysed, both drugs induce a plateau level of F-actin dissociation

and a plateau level of STATc activation. This contrasts markedly with the highly

transitory effects of exposure to DIF-1. Thus there is a temporal correlation, for all

three agents, between their individual kinetics of F-actin dissociation and their ability

to activate STATc. The one unpredicted feature of the drug kinetics data is that the

plateau level of F-actin dissociation for cytochalasin A is significantly higher than for

latrunculin A while, conversely, the plateau level of STATc activation is reproducibly

higher for cytochalasin A. There are several potential explanations for this apparent

non-congruence. There may be a side-effect of one or other of the two drugs or the

explanation may reflect a real physiological difference; we do not know the precise

mechanism of the STATc activation engendered by dissociation of the actin

cytoskeleton and the two drugs act to bring about dissociation by very different

mechanisms.”

*3. cAMP induced F-Actin activation peaks around 10-20 seconds which adapts by 1*

*minute. How does authors exclude that there is no increase in F-Actin level by DIF-1*

*treatment?*

This is a very valid point and we performed the experiments summarised in new Fig

1C to test the possibility. This analysis clearly shows that there is no such increase.

*4. Fig 1B. There is no image for the time zero. Need to show how cells were before*

*the treatments.*

Since these are confocal images we cannot record the exact moment of addition.

Hence we now also show cells at one minute before treatment.

*5. Fig 1B. Cell cringing effect (at 1 and 2 minute) from DIF-1 is less than impressive*

*and there seems to be a second peak at 4 minute.*

The “cringe response” is the immobilization and rounding up of a Dictyostelium cell

in response to cAMP. This is also a totally reproducible aspect of the DIF response;

entire fields of DIF-treated cells, at one to two minutes, will all show the rounding

that is exemplified in Fig 1B. The cringe response is associated with a re-distribution

of F- actin, to populate the cortex uniformly, and this also occurs totally reproducibly

when DIF is added to cells. Actin levels at the cortex are, admittedly, much higher

and more highly maintained after sorbitol treatment but this correlates very well with

sorbitol’s stronger and much more prolonged activation of STATc.

There is no second peak of rounding at four minutes after DIF treatment, rather there

is the typical association of F-actin with stochastically extended lamellipodia.

Reviewer 2

*Figures (or M&M) need to mention how many times experiments have been done;*

These details have now been included in the Figure legends.

*Fig. 1C which mentions that it represents 3 independent experiments needs p values.*

Because of the criticisms of Reviewer 1 we repeated the experiments in Fig 1C and

now present a more comprehensive time course with statistical analyses.

*Despite a convincing p-signal, Fig. 2A needs the loading control (e.g. STAT)*

We have repeated the experiment with a STATc loading control that is now included.

*Fig. 2A and 2B: assuming that all samples in 2A reflect an equal loading, it would*

*appear that despite equivalent amounts of p-STAT in sorbitol and cytochalasin Atreated*

*samples, there is much more nuclear p-STAT in the nucleus after sorbitol*

*treatment than cytochalasin A. This suggests that perturbations in the cytoskeleton*

*are not sufficient for nuclear translocation of STAT, or alternatively, nuclear*

*retention. The authors should include this in the discussion.*

The loading control now included in Fig 2A confirms that sorbitol and cytochalasin

do indeed induce approximately equal amounts of tyrosine phosphorylation of

STATc. As the reviewer correctly points out sorbitol is, nonetheless, a stronger

inducer of STATc nuclear accumulation than cytochalasin A. We now suggest that

stress may signal through a parallel pathway.

P4 line 9 “There is, however, an apparent anomaly with sorbitol, which was included

as a positive control. Sorbitol and cytochalasin induce approximately equal amounts

of tyrosine phosphorylation of STATc but sorbitol is a stronger inducer of STATc

nuclear accumulation than is cytochalasin A. Perhaps, therefore, osmotic stress

signals through an additional, parallel pathway that is independent of tyrosine

phosphorylation to stimulate the nuclear translocation of STATc”.

*There are a few typos in M&Ms and legends that should be corrected*

These are corrected.

**Araki, T., van Egmond, W. N., van Haastert, P. J. and Williams, J. G.** (2010).

Dual regulation of a Dictyostelium STAT by cGMP and Ca2+ signalling. *J Cell Sci*

**123**, 837-41.

**Kortholt, A., Van Egmond, W. N., Plak, K., Bosgraaf, L., Keizer-Gunnink, I. and**

**Van Haastert, P. J.** (2011). Multiple regulatory mechanisms for the Dictyostelium

Roco protein GbpC. *J Biol Chem*. Epub date 2011/11/29

**Lee, E., Shelden, E. A. and Knecht, D. A.** (1998). Formation of F-actin aggregates

in cells treated with actin stabilizing drugs. *Cell Motil. Cytoskel.* **39**, 122-
